# Supplementary material for: Gigahertz optoacoustic vibration in Sub-5 nm tip-supported nano-optomechanical metasurface
Source: Nat Commun. 2023 Jan 30;14:485. doi: 10.1038/s41467-023-36127-6 (PMC9886940; doi:10.1038/s41467-023-36127-6)
Supplement: Supplementary file 1 — Supplementary Information [file 41467_2023_36127_MOESM1_ESM.pdf]

Supplementary information

Gigahertz Optoacoustic Vibration in Sub-5 nm Tip-supported Nano-optomechanical Metasurface

Renxian Gao[#1], Yonglin He[#1], Dumeng Zhang[1], Guoya Sun[1], Jia-Xing He[2] Jian-Feng Li[3], Ming-De Li[*2], Zhilin Yang[*1]

[1] College of Physical Science and Technology, Xiamen University, Xiamen, 361005, China.

[2] Key Laboratory for Preparation and Application of Ordered Structural Materials of Guangdong Province, Department of Chemistry, Shantou University, Shantou, 515063, China.

[3] State Key Laboratory of Physical Chemistry of Solid Surfaces, College of Chemistry and Chemical Engineering, Xiamen University, Xiamen, 361005, China.

[#] These authors contributed equally to this work.

[*] Correspondence to: E-mail: zlyang@xmu.edu.cn; mdli@stu.edu.cn

1. Fabrication of sub-5 nm tip-supported nano-optomechanical metasurface

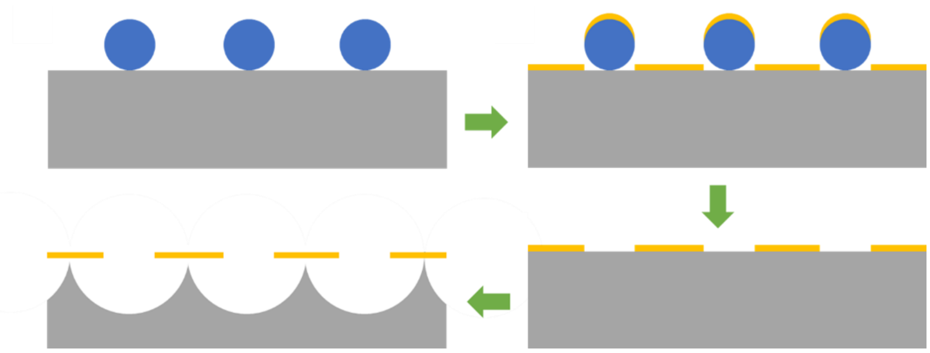

Supplementary Fig. 1 | The process flow of the tip-supported nano-optomechanical metasurface (TSNOMS). TSNOMS was fabricated using the Inductively Coupled Plasma (ICP Sentech, Germany). First, the hexagonally close-packed polystyrene (PS) nanosphere monolayer was assembled on a silicon substrate by an air/water interface method. Then subsequently experienced a reactive ion etching process with a power of 140 W aiming to adjust the diameter of polystyrene spheres. Next, these pre-etched templates were placed in an electric beam evaporation system (aTEMD-500 China), where thin Au films were deposited onto the PS templates under a high vacuum of 9.9×10^{-4} Pa and a deposition rate of 4 Å/s. Gold nanohole arrays were left on the substrates after removing the nanospheres by the polyimide high-temperature resistant tape. Afterward, an inductively coupled plasma (ICP Sentech Germany) etching device was used to etch silicon substrates. ICP etching power is 120 W, SF_6 gas flow rate is 20 sccm, the oxygen flow rate is 10 sccm and C4F8 flow rate is 20 sccm. As the etching time increases, nano-tips are formed underneath the gold nanohole arrays, resulting in the successful preparation of a sub-5 nm TSNOMS.

2. Scanning electron microscope image of TSNOMS

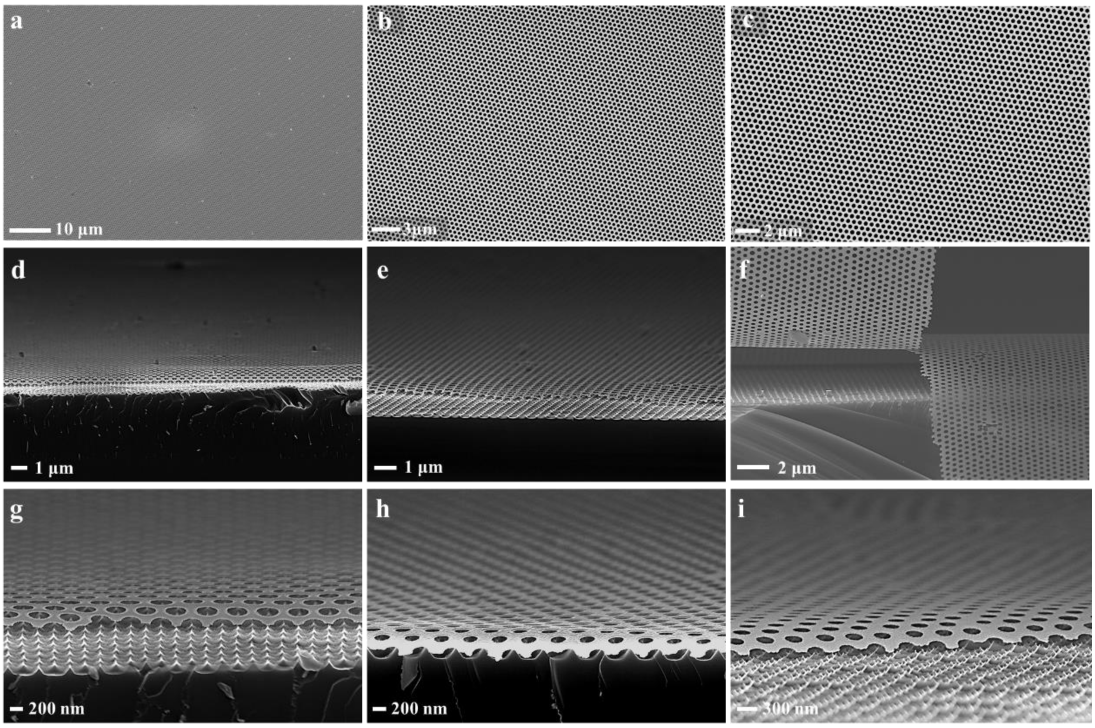

Supplementary Fig. 2 | Scanning electron microscopy (SEM) image of the TSNOMS. a, b, c, d, e, Low magnification SEM images of the TSNOMS, the TSNOMS exhibits a high size homogeneity and alignment in a large area. **f,** The SEM image of the gold nanoholes film after the upper Au film is lifted, which shows the high flexibility of the gold film. **g, h, i,** Cross-section views of the SEM with different hole diameters.

3. Optical finite element simulation of TSNOMS

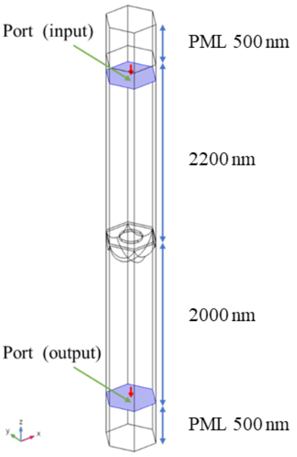

Supplementary Fig. 3 | The geometry of the unit-cell structure is used for electromagnetic simulations. The far-field reflectance spectrum and local-field distributions of the TSNOMS were simulated with a frequency-domain finite-element method to solve the Maxwell equations. The unit cell consists of a silicon substrate, the silicon tips, a gold nano-hole, and the vacuum above them. The uppermost and lowermost regions of the unit cell are defined as the perfectly matched layers (PMLs), which absorb the electromagnetic field. The six transverse boundaries of the unit cell are defined as periodic boundary conditions. The input and output ports are defined in the purple plane.

Approximately 120,000 tetrahedral mesh elements are used for the discretization, with a maximum size of 70 nm in the vacuum and the silicon substrate and a maximum of 5 nm in the gold nano-hole. A finer mesh was used near the corners of the silicon tips.

4. Model analysis of interband transition absorption induced by ultraviolet interference

Supplementary Fig. 4a shows the experimental and simulated reflectance spectra of the TSNOMS (including the UV band). In the main manuscript, the deep at 311 nm was attributed to the interband transition absorption of gold. To ensure the accuracy of these speculations, wave optics theory and numerical finite element simulation were used to describe them in detail. When a beam of light is reflected by the substrate, the reflected light is superimposed on the incident light to form a standing wave.

Incident wave:

$$\mathbf{E}_{in}=\mathbf{E}_0 e^{i(\mathbf{k}z-\omega t)}$$

Reflected wave:

$$\mathbf{E}_r=r\mathbf{E}_0 e^{i(-\mathbf{k}z-\omega t)}$$

The form of the field of the superimposed incident and reflected light is as follows:

$$\mathbf{E}=\mathbf{E}_0 e^{i(\mathbf{k}z-\omega t)}+r\mathbf{E}_0 e^{i(-\mathbf{k}z-\omega t)}$$

$$\mathbf{E}=\mathbf{E}_0[(1+r_1)\,cos(\mathbf{k}z)+ir_2\,cos(\mathbf{k}z)+i(1-r_1)\,sin(\mathbf{k}z)+r_2\,sin(\mathbf{k}z)]\,[cos(\omega t)-i\,sin(\omega t)]$$

After retaining the real part:

$$\mathbf{E}=\mathbf{E}_0\sqrt{(1+|r|^2)+2|r|\,cos(2\boldsymbol{k}z+\varphi_1)}\,sin(\omega t+\varphi)$$

The above equation shows that the electric field intensity is a function of spatial position z, and the distance between the antinode and the node of a standing wave is $\Delta z=\lambda/4$. According to the above derivation combined with the reflection spectrum shown in Supplementary Fig. 4a, the distribution of the metasurface electric field was calculated by the finite element method when the incident wavelength was 215, 311, 257, and 434 nm (Supplementary Fig. 4b). As shown in Supplementary Fig. 4b, the distance between the electric field ventral and the electric field nodes is consistent with the above corollary. Due to the support of the silicon nanotips, when the incident light wavelength is 215 or 311 nm, the diffraction wave junction covers the plane where the gold metasurface is located. When the incident wavelength is 257 or 434 nm, the distance between the electric field node and the gold metasurface is exactly one-eighth of the wavelength. Therefore, when the incident light energy is higher than the interband transition energy of gold, the more overlap between the electric field node and the gold metasurface, and the higher absorption rate of the metasurface. Based on the above discussion, the absorption dip at a wavelength of 311 nm is confirmed to originate from the interband transition absorption of gold.

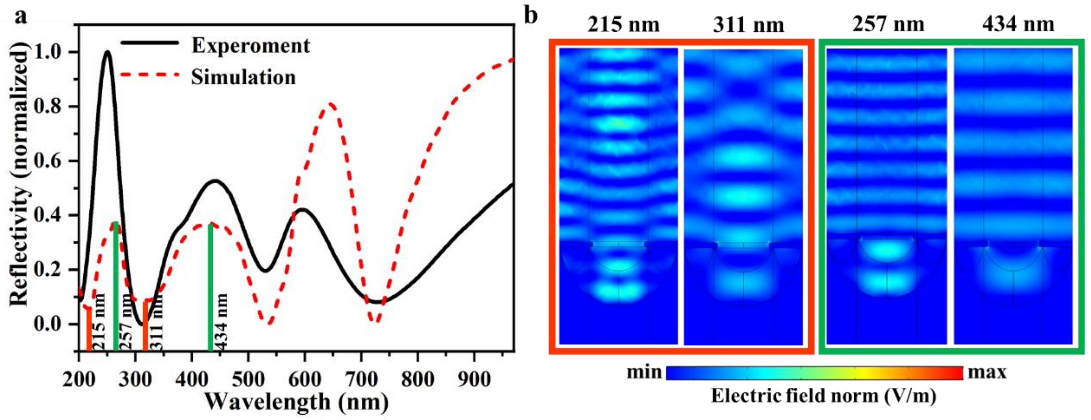

Supplementary Fig. 4 | The simulated reflection spectra and electric field intensity distribution of the TSNOMS. a, The measured reflection spectra of the TSNOMS (black lines), and the simulated reflection spectra calculated by COMSOL (red dotted line). **b,** The simulated electric field intensity distribution of the TSNOMS.

5. Schematic diagram of the pump-probe experiment

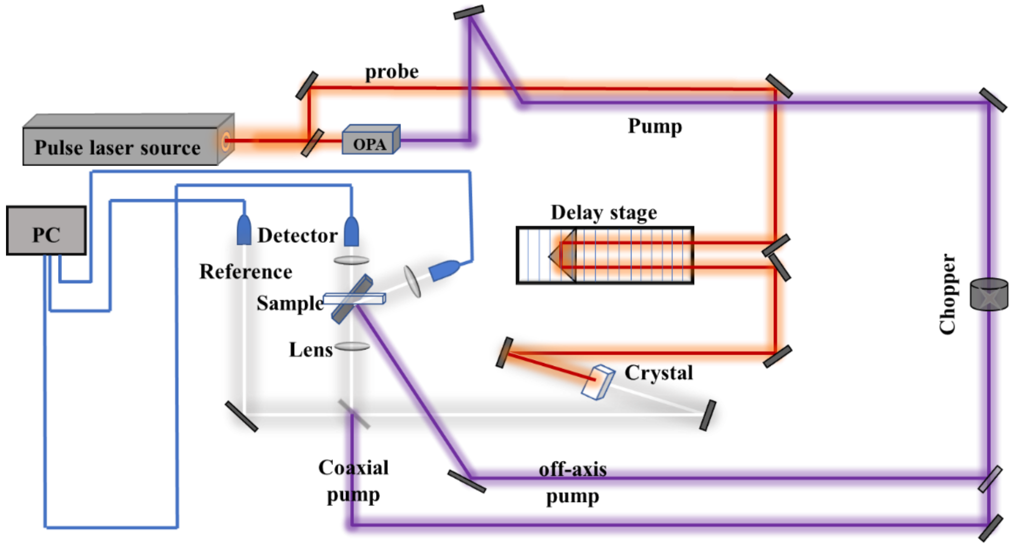

Supplementary Fig. 5 | Schematic diagram of the pump-probe experiment. Schematic showing the ultrafast pump-probe setup.

6. Structure vibration main time domain processes driven by a femtosecond pump pulse

In this approach, the 311 nm pump pulse was used to selectively excite the conduction electrons of the metal by interband transition absorption, followed by fast Auger relaxation of the photoexcited electrons[2] (see step 1 in Supplementary Fig. 6). Except for very short time delays, the excitation processes lead to similar nonequilibrium conduction electron distribution that thermalizes by electron-electron scattering[3] (see step 2 in Supplementary Fig. 6). The energy given to the electrons is subsequently damped to the lattice by electron-vibration interactions (see step 3 in Supplementary Fig. 6). The time evolutions of the electronic T_e and lattice T_l temperatures are described by the rate equation system (two-temperature model)[4-6]:

$$C_e(T_e)\frac{dT_e}{dt}=\nabla\cdot(\kappa_e \nabla T_e)-G(T_e)(T_e-T_l)+S(\mathbf{r},t)$$

$$C_l\frac{dT_l}{dt}=\kappa_l\nabla^2 T_l+G(T_e)(T_e\text{-}T_l)$$

Excitation of the metal electrons and electron-lattice thermalization leads to hot nano-objects that cool down to initial temperature by energy transfer to their surroundings (matrix or substrate) and heat diffusion in the latter[7]. This takes place on a timescale of typically a few tens to hundred picoseconds depending on the environment, the size of the object, and their coupling. Direct electron heating by the pump pulse and fast indirect heating of the lattice by electron-lattice energy transfer both impose dilation of the nano-object and launch its acoustic vibrations[8] (see step 4 in Supplementary Fig. 6). Heating of the electrons and lattice of a nano-object and launching of its acoustic vibration modify its dielectric function. This translates into a time-dependent modification of its optical response that can be followed using femtosecond transient spectroscopy.

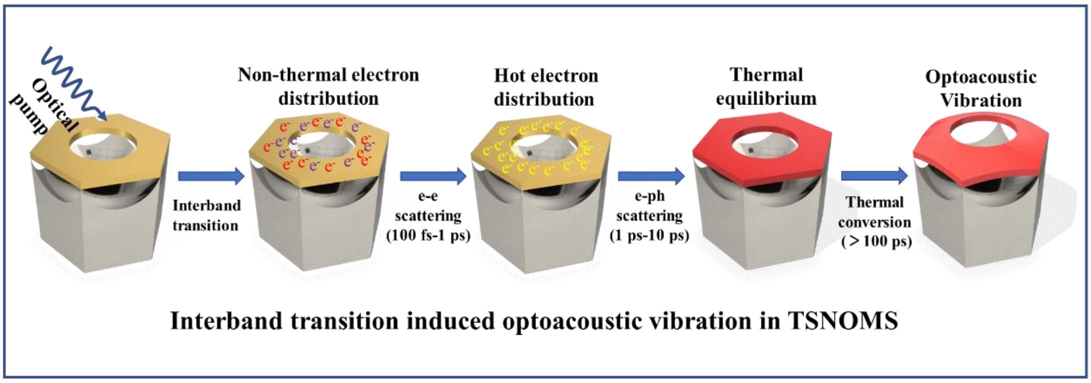

Supplementary Fig. 6 | Structure vibration main time domain processes. Main time-domain processes after selective electron heating at temperature of the electron gas of the TSNOMS by a femtosecond pulse: electron-lattice thermalization, acoustic vibrations, and thermal and acoustic energy damping to the environment.

7. The reflectance spectrum of the structure after vibration deformation

To further confirm the accuracy of the mechanical oscillation mode attribution, The finite element method was used to qualitatively analyze the reflection spectrum of the structure after oscillating deformation. Firstly, the reflectance spectra of TSNOMS with nanohole diameters R=250 nm and R=250±10 nm was calculated separately (the structure diagram and simulation structure diagram are shown in Supplementary Fig. 7a, b). Supplementary Fig. 7c shows the simulation results for the reflection spectra of a TSNOMS before and after the in-plane deformation. As shown in Supplementary Fig. 7c, the variation in the hole diameter of the TSNOMS has a pronounced modulation effect on the red and blue sidebands of the localized surface plasmon resonance (LSPR) mode, which is in perfect agreement with the LSPR red and blue sideband modulation results shown in Supplementary Fig. 7d of the previous manuscript. Therefore, the above simulation results demonstrate that the high-frequency oscillations of the transient signal in the red and blue sidebands of the LSPR mode are caused by modulation of the spectral dispersion by the periodic in-plane deformation of the nanohole.

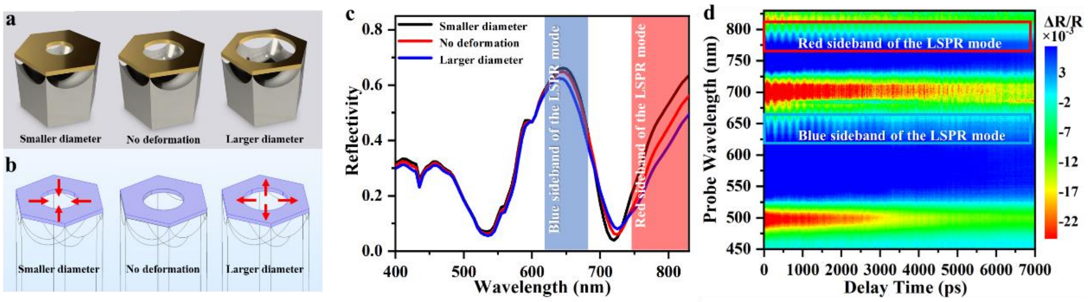

Supplementary Fig. 7 | Structure diagram and reflectance spectrum of the structure after vibration deformation. a, The structure diagram of in-plane deformations. **b,** Simulation structure diagram. **c,** Simulate the reflectance spectra of in-plane deformations. **d,** Transient reflection spectral map of the TSNOMS for the spectral range between 450 and 830 nm with a delay time up to 7000 ps.

Subsequently, the reflectance spectra of the nanohole deformation along the Z direction was calculated as Δu=±10 nm and Δu=0 nm, respectively. The structure diagram and simulation structure diagram are shown in Supplementary Fig. 8a, b. Supplementary Fig. 8c shows the simulation results for the reflection spectra of the TSNOMS before and after the out-of-plane deformation. As shown in Supplementary Fig. 8c, the out-of-plane periodic oscillation mode modulates both the red and blue sidebands of the propagating surface plasmons (PSPs) resonance. In contrast, the modulation is present only on the red sideband of the PSPs resonances in Supplementary Fig. 8d. To investigate the reasons for the conflicting results, we performed the transient dynamic signals of gold nanofilms (Supplementary Fig. 8e). In Supplementary Fig. 8e an obvious transient dynamic signal of the interband transition of gold appears at approximately 510 nm. Therefore, we can determine that there is energy competition between the interband transition of gold in the TSNOMS and the PSPs resonance. As a result, the transient signal in the blue sideband of the PSPs resonance is covered by the transient dynamic signal of the interband transition of gold. Therefore, the above results demonstrate that the relatively low-frequency oscillations of the transient signal in the red sideband of the PSPs mode are caused by modulation of the spectral dispersion by the periodic out-of-plane deformation of the metasurface.

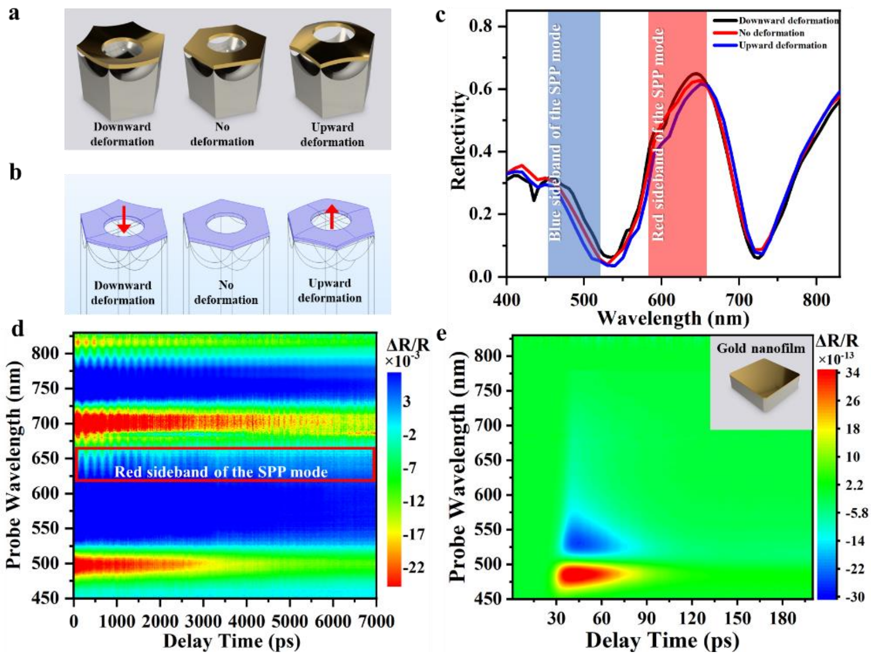

Supplementary Fig. 8 | Structure diagram and reflectance spectrum of the structure after vibration deformation. a, The structure diagram of out-of-plane deformations. **b,** Simulation structure diagram. **c,** Simulate the reflectance spectra of out-of-plane deformations. **d,** Transient reflection spectral map of the TSNOMS for the spectral range between 450 and 830 nm with a delay time up to 7000 ps. **e,** Transient reflection spectral map of the gold nanofilm.

8. Power-dependent transient reflection spectra

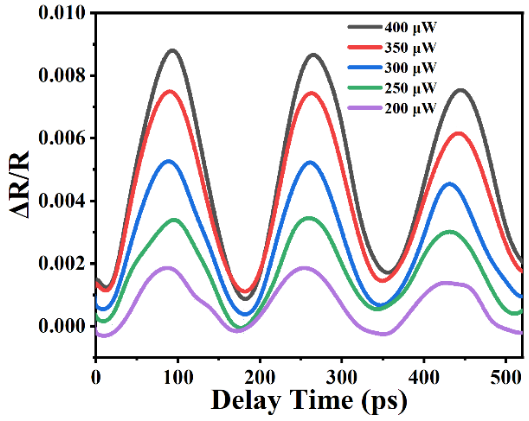

Supplementary Fig. 9 | Power-dependent transient reflection spectra.

9. Numerical calculation of photothermal conversion, thermal energy, and mechanical energy losses for metasurfaces with different nanotip sizes

The conversion of light energy to mechanical energy in an optomechanical system consists of three main energy loss channels: photothermal, structural thermal, and mechanical energy losses. To illustrate the meaning of "the energy losses caused by the substrate to be infinitely close to the theoretical limit" in the previous manuscript, we calculated the photothermal conversion, thermal energy, and mechanical energy losses for metasurfaces with different contact areas with the substrate using a two-temperature model and the finite element method (see Supplementary Fig. 10). In terms of photothermal conversion loss, we calculated the variation in electron and lattice temperatures over time at the metasurface under 311-nm laser excitation using the wave optics model of COMSOL and the two-temperature model equation (Methods). Supplementary Fig. 10a shows that the four structures have different quasi-equilibrium temperatures when the electron and phonon temperatures converge due to the different coupling capabilities of the four structures to the 311 nm laser. Supplementary Fig. 10b shows that as the substrate contact area decreases, the electron-lattice quasi-equilibrium temperature gradually increases, and it reaches a maximum when the nanotips are less than 5 nm wide. This means that the sub-5 nm nanotip array allows the TSNOMS to more easily couple the energy of the pulsed laser into the optomechanical system to excite optoacoustic vibration. In terms of structural thermal energy loss, the thermal distributions in the time domain of the four supported forms of the metasurface were calculated by coupling the solid-state heat transfer model. Supplementary Fig. 10c, d shows that the sub-5 nm nanotips of Structure 4 minimize the thermal loss from the substrate compared to Structures 1, 2 and 3. For the mechanical energy channel, the geometric strain of the metasurface under the same prestress was simulated by the structural mechanics model for both support methods (see Supplementary Fig. 10e, f). The sub-5 nm tip-supported metasurface (Structure 4) has the highest strain rate for the same prestress

conditions, which indicates that less than the sub-5 nm tip-supported metasurface has the lowest mechanical energy loss for the same pulse stress. Therefore, the numerical theoretical analysis of the three energy loss channels determined that the design strategy of a sub-5nm tip-supported metasurface can reduce the energy loss from the substrate to close to the theoretical limit. The multichannel-loss-mitigating semi-suspended metasurface design strategy can be generalized to performance improvements of most on-chip processed nano-optomechanical systems.

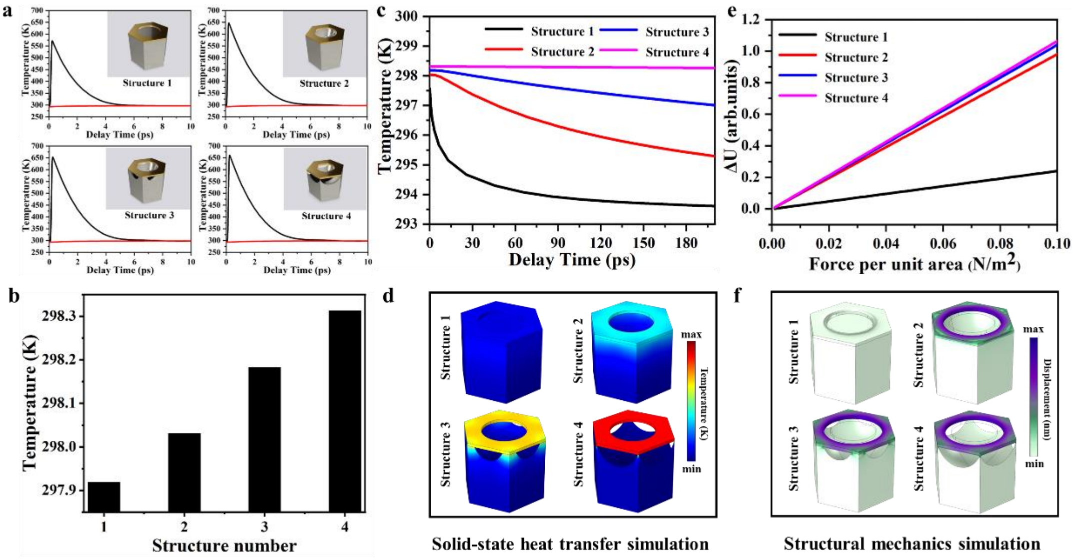

Supplementary Fig. 10 | Photothermal conversion, thermal energy and mechanical energy simulations. a, Simulate the electronic and lattice temperature of four supported forms of metasurface by calculating the solid-state heat transfer module with two temperature model (2TM) and the wave optics module of COMSOL. **b,** electron-lattice quasi-equilibrium temperature of four supported forms of metasurface. **c, d,** Simulated thermal distribution in the time domain of the four supported forms of the optomechanical metasurface. **e, f,** The simulated geometric strain of the metasurface under the same prestress.

10. Uniformity test

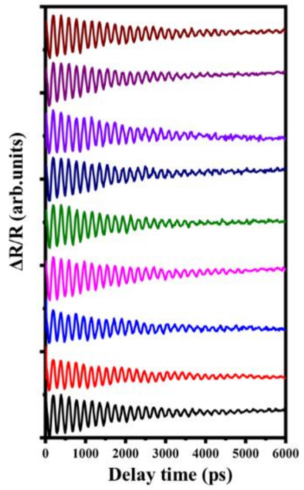

Supplementary Fig. 11 | Time-domain plots corresponding to the indicated scan regions are stacked.

Supplementary References

1. Cooper B.R., Ehrenreich H., Philipp H.R. Optical Properties of Noble Metals. II. *Phys. Rev.* **138**, A494 (1965).

2. Knoesel E. et al. Ultrafast dynamics of hot electrons and holes in copper: Excitation, energy relaxation, and transport effects. *Phys. Rev. B* **57**, 12812-12824 (1998).

3. Voisin C. et al. Size-dependent electron–electron interactions in metal nanoparticles. *Phys. Rev. Lett.* **85**, 2200-2203 (2000).

4. Block A. et al. Tracking ultrafast hot-electron diffusion in space and time by ultrafast thermomodulation microscopy. *Sci. Adv.* **5**, 8965 (2019).

5. Brown A. M. et al. Ab initiophonon coupling and optical response of hot electrons in plasmonic metals. *Phys. Rev. B* **94**, 075120 (2016).

6. Schirato A. et al. Transient optical symmetry breaking for ultrafast broadband dichroism in plasmonic metasurfaces. *Nat. Photon.* **14**, 723-727 (2020).

7. Juvé V. et al. Cooling dynamics and thermal interface resistance of glass-embedded metal nanoparticles. *Phys. Rev. B* **80**, 195406 (2009).

8. Aurelien C. et al. Acoustic vibrations of metal nano-objects: Time-domain investigations. *Phys. Rep.* **549**, 1-43 (2015).
